# Epidemiology of *Theileria bicornis* among black and white rhinoceros metapopulation in Kenya

**DOI:** 10.1186/s12917-014-0316-2

**Published:** 2015-01-17

**Authors:** Moses Y Otiende, Mary W Kivata, Joseph N Makumi, Mathew N Mutinda, Daniel Okun, Linus Kariuki, Vincent Obanda, Francis Gakuya, Dominic Mijele, Ramón C Soriguer, Samer Alasaad

**Affiliations:** Veterinary Services Department, Forensic and Genetics Laboratory Kenya Wildlife Service, P.O Box 40241–00100, Nairobi, Kenya; Estación Biológica de Doñana, Consejo Superior de Investigaciones Científicas (CSIC), Avda. Américo Vespucio s/n 41092, Sevilla, Spain; Institute of Evolutionary Biology and Environmental Studies (IEU), University of Zürich, Winterthurerstrasse 190, 8057 Zürich, Switzerland; Department of Biochemistry and Biotechnology, Kenyatta University, P.O Box 43844–00100, Nairobi, Kenya

**Keywords:** Ixodid, Ticks, Piroplasms, Diceros bicornis michaeli, Ceratotherium simum simum

## Abstract

**Background:**

A huge effort in rhinoceros conservation has focused on poaching and habitat loss as factors leading to the dramatic declines in the endangered eastern black rhinoceros (*Diceros bicornis michaeli*) and the southern white rhinoceros (*Ceratotherium simum simum*). Nevertheless, the role disease and parasite infections play in the mortality of protected populations has largely received limited attention. Infections with piroplasmosis caused by *Babesia bicornis* and *Theileria bicornis* has been shown to be fatal especially in small and isolated populations in Tanzania and South Africa. However, the occurrence and epidemiology of these parasites in Kenyan rhinoceros is not known.

**Results:**

Utilizing 18S rRNA gene as genetic marker to detect rhinoceros infection with *Babesia* and *Theileria*, we examined blood samples collected from seven rhinoceros populations consisting of 114 individuals of black and white rhinoceros. The goal was to determine the prevalence in Kenyan populations, and to assess the association of *Babesia* and *Theileria* infection with host species, age, sex, location, season and population mix (only black rhinoceros comparing to black and white rhinoceros populations). We did not detect any infection with *Babesia* in the sequenced samples, while the prevalence of *T. bicornis* in the Kenyan rhinoceros population was 49.12% (56/114). White rhinoceros had significantly higher prevalence of infection (66%) compared to black rhinoceros (43%). The infection of rhinoceros with *Theileria* was not associated with animal age, sex or location. The risk of infection with *Theileria* was not higher in mixed species populations compared to populations of pure black rhinoceros.

**Conclusion:**

In the rhinoceros studied, we did not detect the presence of *Babesia bicornis*, while *Theileria bicornis* was found to have a 49.12% prevalence with white rhinoceros showing a higher prevalence (66%) comparing with black rhinoceros (43%). Other factors such as age, sex, location, and population mix were not found to play a significant role.

## Background

The populations and distribution ranges of the black rhinoceros (*Diceros bicornis*) and the white rhinoceros (*Ceratotherium simum*) have declined in the whole of Africa. The rate of their population decline is faster than any other large terrestrial mammal in recent times [1], a fact that supports their endangered status and calls for robust international efforts towards their recovery. These rhinoceros have been exterminated in the majority of African countries, while their range among the remaining principal countries; Kenya, Tanzania, Namibia, Zimbabwe and South Africa [1] is greatly reduced and currently restricted in artificially created sanctuaries. Habitat loss and vicious poaching are the leading twin drivers of population decline of the rhinoceros [2]. However infectious diseases are also an incipient threat to endangered species [3] having been classified among the top five causes of species extinctions [4].

Piroplasms, which are blood-borne protozoan parasites in the genera *Babesia* and *Theileria* (Order Piroplasmidae), are globally distributed and transmitted by a diverse species of Ixodid ticks. These parasites infect a wide range of domesticated and wild mammals as well as humans. Infections may lead to severe disease and death or it may remain latent depending on virulence of the species and host immune status. Piroplasms have historically been known to infect rhinoceros with some infections associated with fatalities [5-7]. However the causal species were unknown until 10 years ago when *Babesia bicornis* and *Theileria bicornis* were independently associated with stress-induced mortality [8]. The first genetic work on piroplasms by Otiende et al., [9] has shown the existence of infection by piroplasm and the occurrence of three new haplotypes of *Theileria bicornis* circulating in both black and white rhinoceros in Kenya. The factors influencing piroplasms prevalence among Kenyan populations were previously unknown. Piroplasms have coevolved with rhinoceros and they coexist with the host without signs of clinical disease. However, stress induced by translocation has been linked to immune suppression and is a major cause of post translocation morbidity and/or fatality. Translocation is at the core of *in situ* management of rhinoceros metapopulation and yet it is incriminated as a disease inducer besides its inherent role in the spread of pathogens. The link between translocation and piroplasmosis is intricate because it is based on the modulatory effects of stress hormones on the immune system. Translocation elicits stress hormones, which allow uninhibited proliferation of piroplasms in the host resulting in disease and death. However, effects of stress hormones are not predictable or homogenous in the population as underlying individuals’ conditions, such as injury, pregnancy, co-infection, vary and may elicit different immune response [10].

The goal of this study is to determine the epidemiology of *Theileria biconis* in Kenya. Specifically, we intended to (a) determine their prevalence in both species of rhinoceros and among sub-populations then (b) test the association of infection prevalence with host species, age, sex, location, season and population mix (black rhinoceros vs black and white rhinoceros populations). Information generated will be useful in guiding management and veterinary options such as translocation, differential diagnosis and chemotherapy.

## Methods

### Study area

*Lake Nakuru National Park* (*LNNP*) central coordinates are 0°22′S 36°05′E and is 4 km from Nakuru town center. The park covers an area of 188 km^2^ completely fenced, of which 44 km^2^ lies in the shallow alkaline soda lake, thereby leaving 144 km^2^ for wildlife use. The area around the lake is flat bare lowland of 1200 m altitude surrounded by hills and gentle cliffs that rise to 1750 m above sea level. The park receives mean annual rainfall of 850 mm with rainfall in the months of April to May and again in October – November. The park consists of open grassland with elevated areas occupied by dry forests of *Acacia xanthophloea*, *Olea capensis* sub sp. *Macrocarpa* and *Croton dichogamus*. Marshland along the river inflows and springs are covered by *Cyperus laevigatus and Typha spp*. Other striking plant species include the invasive *Tarchonanthus* spp. bush land, the deciduous (*Teclea & Olive*) forest and the *Euphorbia candelabrum* forest. The park has 33 white and 69 black rhinoceros that freely interact with other diverse species.

*Nairobi National Park* (*NNP*) central coordinates are 1°16′S, 36°49′E and is about 10 km from the city of Nairobi and covers an area of 117 km^2^. A large section of the park is fenced with only 20 km left open for wildlife dispersal. Average annual rainfall is 800 mm with rainy season between April-May and October-November. The vegetation consists of mosaic grassland, thickets and Acacia and deciduous forests as well as woodlands especially along River Mbagathi that crosses it. The park has 77 black and 13 white rhinoceros besides many other wildlife species.

*Ngulia rhino Sanctuary* (*NgRS*) is within Tsavo West National Park occupying a fenced area of 90 km^2^ at 3^0^ 01′S to 3^0^ 06′S and 38^0^ 06E to 38^0^ 10′E. Altitude ranges from 600 m of lowlands to 1800 m of craggy hills with average annual rainfall of 600 mm. Dry period is between December to March, while rains occur in the months of April to June and again in October and November. The vegetation is thickly wooded by *Commiphora*-*Acacia* woodland, dotted with baobab trees. This sanctuary contains 77 black rhinoceros without white rhinoceros, though other small-medium sized wildlife occurs in small density.

*Meru Rhino Sanctuary* (*MRS*) is 48Km^2^ (central coordinates, N 00^0^ 15.125, E 038^0^06.481) located within the Meru National Park has 44 black and 45 white rhinoceros. Altitude ranges from 1000 to 3400 m above the sea level. Average rainfall is 635-762 mm with the wet season occurring in late March to April, while the dry season begins from October. Major rivers such as Makutano, Kanjoo, Kathithi, Rujuwero and Kindani traverse MRS, which contributes to mosaic vegetation types that include thickets, bushland and grassland as well as a thick forest on its southern edge.

*Solio and Mugie Rhino Sanctuaries* are in the Laikipia-Samburu ecosystem, which is characterized by savannah-type grassland dominated by *Euclea divinorum*, *Acacia spp and Euphorbia* woodland while annual rainfall averages 300 to 700 mm. Rainfall level varies annually with intermittent patterns that peaks in April-May, July–August and October–November. The rest of the months are dry. Solio Sanctuary, 0°16′S 37°00′E / 0.27°S 37°E/ -0.27; 37, is 68.9 km^2^ and has altitude of 1932 m, located at the base of the Aberdare ranges. The sanctuary holds 50 black and 110 white rhinoceros. Mugie rhino sanctuary (ceased to be a rhino sanctuary at the time of this study, as the entire population was translocated) was 90 km^2^ with an altitude 1990 m.

### Sampling design

This study was carried out between 2011 and 2012 in various sub populations of black and white rhinoceros in Kenya. Sampling was cross-sectional whereby samples were collected from apparently healthy rhinos immobilized for translocation or tagging. The biodata of each rhinoceros was obtained from the KWS rhinoceros database. Rhinoceros < 2 years of age were not immobilized for translocation. Juvenile rhinoceros are <3.5 years, sub-adults are (3.6 – 7 years) and adults are >7 years. Rhinoceros were immobilized using a combination of etorphine hydrochloride (M99®), Hyaluronidase (Kyron Laboratories, Benrose 2011, South Africa) and xylazine (Norvatis, [PTY] Ltd, South Africa). Venous blood was drawn from the front limb of the rhinoceros and then collected in EDTA tubes. Blood in EDTA tubes were gently mixed by turning the tubes up and down and then transferring aliquots in labeled cryovials followed by quick freezing in liquid nitrogen. The samples were transported in liquid nitrogen to Forensic and Genetics Laboratory of Kenya Wildlife Service in Nairobi for analysis.

### DNA isolation and PCR amplification

Genomic DNA was extracted from blood using a genomic DNA extraction kit (DNeasy blood and Tissue Kit, QIAGEN, Southern Cross Biotechnologies, South Africa) following the manufacturers’ protocol. A nested PCR amplification specific for the 18S rRNA gene of *Babesia* and *Theileria* was performed. A primary amplification was carried out in 50 μl reaction containing 3 μl of the genomic DNA, 45 μl of Platinum blue supermix, 1 μl (10 mM) each forward and reverse primers. The forward primer was ILO-9029, (5′-CGGTAATTCCAGCTCCAATAGCGT-3′) and reverse, ILO-9030 (5′-TTTCTCTCAAAGGTGCTGAAGGAGT-3′) primer [11]. The amplification (Thermocycler, Veriti, ABI) was preceded by a 30 sec polymerase activation step at 95°C followed by 30 cycles of 1 min each at 94°C, annealing at 53°C for 30 sec, extension for 1 min at 72°C. Amplification was terminated by a final extension step 72°C for 9 min. The secondary amplification was in a 50 μl reaction containing 2 μl of the primary amplification product, 45 μl of Platinum blue supermix, 1.5 μl (10 mM) each of forward and reverse primers. The forward primer was MWG4/70, (5′-AGCTCGTAGTTGAATTTCTGCTGC-3′) and the reverse was ILO-7782 (5′-AACTGACGACCTCCAATCTCTAGTC-3′) [11]. The secondary PCR (Thermocycler, Veriti, ABI) was initiated with an initial denaturation at 95°C for 30 sec, followed by 30 cycles of 1 min each at 94°C, annealing at 55°C for 30 s and extension at 72°C for 1 min. The PCR was completed with a final extension step of 72°C for 9 min. PCR products showing successful amplification on agarose gel analysis were directly sequenced for both strands. PCR products were purified for direct sequencing by enzymatic treatment using exonuclease I and shrimp alkaline phosphatase (PCR Product Presequencing Kit, Amersham). All DNA sequencing was carried out by direct cycle sequencing on both strands of purified PCR DNA products from PCR amplification. Sequencing reactions were carried out with the ABI PRISM DigDye Terminator v3.1 cycle sequencing kit and analyzed on an ABI310 DNA sequencer (Applied Biosystems, CA).

### Statistical analysis

Statistical analyses were performed using Fisher Exact test for count data and Chi Square test to determine the relationship between infection of rhinoceros with *Theileria* and the following variables: rhinoceros age, location, sex, and rhinoceros species. To confirm our results, we also used a Generalized Linear Model with a binomial error, and a complementary log-log link function. All possible interactions were included in the first model. As rhinoceros species are essentially clustered with locality (game park or sanctuary) a generalized linear mixed effect model was also applied, considering locality (game park or sanctuary) as random effect: *Theileria* Infection ~ Rhinos Species + (1 | Locality). Statistical significance was assessed at *p* < 0.05.

### Ethic

The Committee of the Department of Veterinary and Capture Services of the Kenya Wildlife Service (KWS) approved the study including animal capture, translocation and sample collection. KWS guidelines on Wildlife Veterinary Practice-2006 were followed. All KWS veterinarians were guided by the Veterinary Surgeons Act Cap 366 Laws of Kenya that regulates veterinary practice in Kenya.

## Results

**Figure 1:**
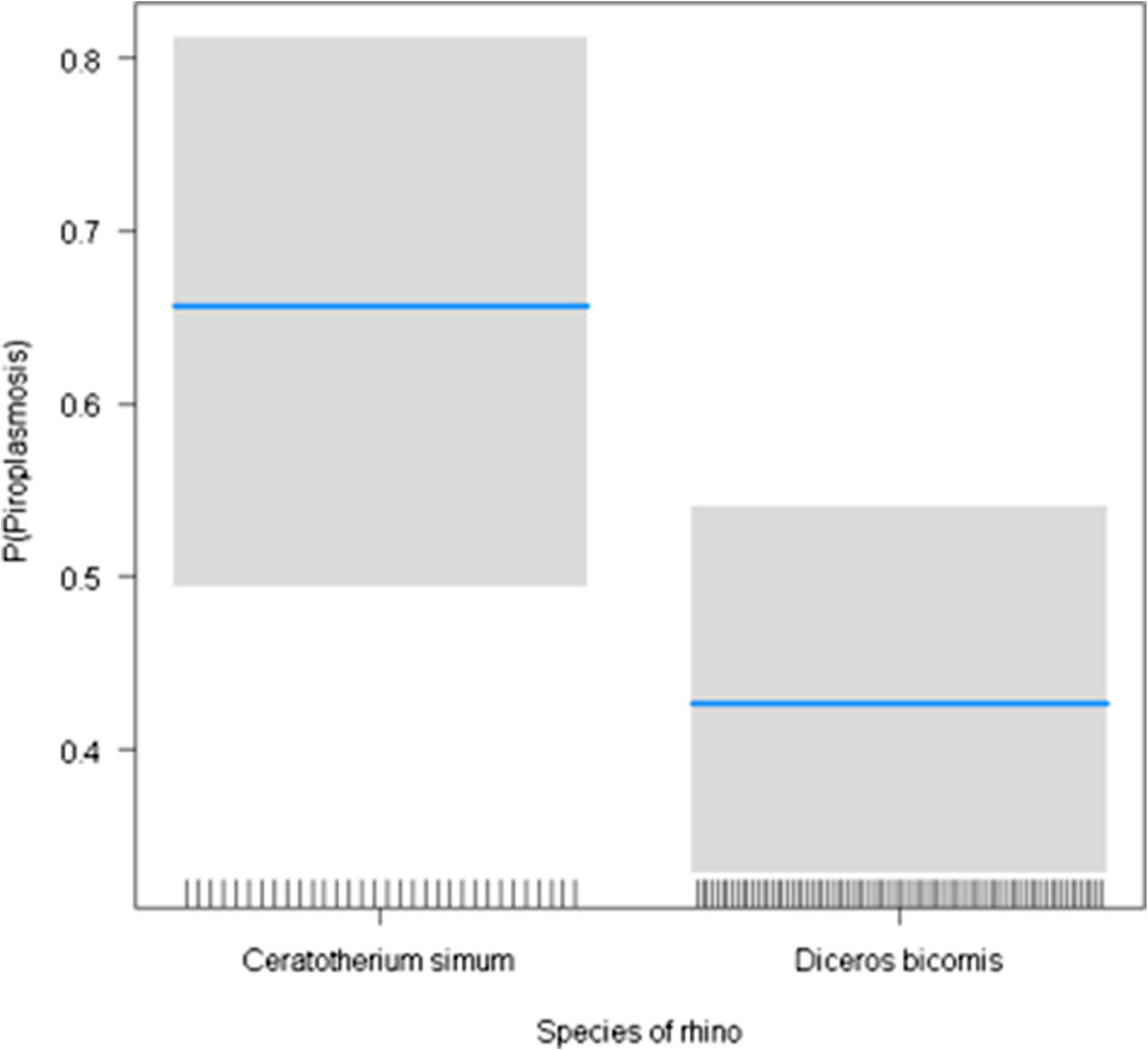
**Variation of infection intensity of rhinoceros by species.**

A total of 114 blood samples of black (*n = 82*) and white rhinoceros (*n = 32*) were sampled from seven rhinoceros populations and molecularly examined for infection with *Babesia* and *Theileria*. We did not detect any infection with *Babesia* in the obtained sequences, while the overall *Theileria* prevalence was 49.1% (56/114). All *Theileria* sequences belonged to the three haplotypes already described by Otiende et al., [9]. The prevalence of *Theileria* infection was higher in white rhinoceros (66%) than in black rhinoceros (43%) (Table 1). We confirmed this result using a generalized linear model (b = −0.652, *p* = 0.023). The simplified glm model was *Theileria* Infection ~ Rhinos Species, family = binomial (cloglog): *Theileria* Infection ~ 0.0656 ± 0.2287 Rhinos Species - 0.6516 ± 0.2857. Since prevalence was higher in white rhinoceros, we tested whether presence of white rhinoceros together with black rhinoceros in the same locality was a risk factor for infection with *Theileria* in black rhinoceros. We found no significant association between infection of black rhinoceros with *Theileria* and the presence or absence of white rhinoceros in the same locality (χ^2^ = 0321; *p* = 0.571). The results were confirmed by applying a generalized lineal mixed effect model, considering locality (game park or sanctuary) as random effect. The last model in this case was *Theileria* Infection ~ Rhinos Species, family = binomial (logit): *Theileria* Infection ~ 0.0647 ± 0.3722 - 0.9414 ± 0.434 (Rhinos Species). Prevalence seemed to increase with age, but the infection by age-groups (juveniles, sub-adults and adults) was not statistically significant (Fisher test, *p* = 0.764, Table 1). Females of both species had higher prevalence 54% (29/54) than males 45% (27/60), this difference however was not statistically significant (Fisher test, *p* = 0.702, Table 1). Inter-population variations in prevalence (Figure 1) were not statistically different (Fisher test, *p* = 0.681, Table 1).

**Table 1:** **Proportion of rhinoceros infected as a function of age, location, sex and species evaluated using Fisher’s exact test**

**Variable**	**Variable categories**	**% Negative**	**% Positive**	**N**	**p-value**
**AGE**	Juvenile	58.8	41.2	17	0.764
	Sub-Adult	48.2	51.8	56	
	Adult	51.2	48.8	41	
**LOCATION**	LNNP	46.7	53.3	35	0.559
	Meru N. P.	50.0	50.0	12	
	Mugie	57.9	42.1	20	
	Ngulia	55.2	44.8	29	
	NNP	55.6	44.4	10	
	Solio	87.5	12.5	8	
**SEX**	Female	41.7	58.3	59	0.7026
	Male	30.0	70.0	55	
**SPECIES**	*C. simum*	34.4	65.6	32	**0.037**
	*D. bicornis*	57.3	42.7	82

## Discussion

The six sampled rhinoceros sub-populations in Kenya were infected with piroplasms but we molecularly detected only *Theileria* and not *Babesia* in all studied samples from black and white rhinoceros species.

Ticks and wildlife are the maintenance hosts of piroplasms but efficient transmission fundamentally requires presence of the protozoan, a competent tick species and the host.

Even with *B. bicornis* and *T. bicornis* originally identified from black rhinoceros [8] *T. bicornis* has now been detected in white rhinoceros, Nyala (*Tragelaphus angasii*) and Cattle [12-14]. This means that *T. bicornis* is a multi-host pathogen with possibility of having diverse tick species as vectors. Since *D. rhinocerinus* have been found in other animals such as cattle, sheep, donkey, elephant (*Loxodonta africana*), buffalo (*Syncerus caffer*) and eland (*Taurotragus oryx*) [15,16] this tick could be important in the cross-transmission of rhinocerotid piroplams. Pathogenicity of *T. bicornis* remains unresolved but *T. equi,* which is its close relative [8,17] and recently seen in white rhinoceros [13], has been reported to cause clinical piroplasmosis in translocated equids [18].

Translocation is intimately associated with flare up of latent infections that result in clinical state [18-20]. This is because translocation leads to elevation of glucocorticoids, whose effects are viewed to be obligatorily immunodepressant [10], yet in most cases, especially in transient acute stress, they prepare an animal to survive [10,21,22]. In the present study, sampling was carried out on asymptomatic individuals and despite underlying infection with *T. bicornis*, some of them were subjected to a longer period of stressor condition; during >1000 km road transportation to a new sanctuary in Ruma National Park. Nevertheless, for six months post-release monitoring of this population, they remained asymptomatic. This outcome may support the notion that *T. bicornis* is apathogenic or it may suggest that translocation-stress did not suppress immunity to induce clinical state. Theories behind disease induction by translocation-stress often focus on single parasite infections. However, in nature, wild animals are infected and infested simultaneously with a plethora of parasites that elicits complex immune response that may promote one parasite over the other. For instance, in concomitant infection involving African trypanosome superimposed with piroplasm leads to inhibition of the piroplasm in spite of the trypanosome immunodepressant effect [23]. In reference to fatal piroplasmosis [8], the deceased rhinoceros were subject to diverse and combined stressors; two black rhinoceros in Tanzania did not undergo prior capture and translocation event; the third fatal case involved high parasitemia, severe cold and injury while the fourth case was pregnant and developed translocation myopathy. This suggests that stressor factors that trigger clinical disease are many with maximum effect attained under synergistic state.

In the present study, the prevalence of *T. bicornis* was relatively high (49.1%) but clinical disease was absent in the metapopulation, a state that could mimics endemic stability [24]. This state, which was initially coined for bovine babesiosis and now widely applied in many diseases and hosts, is based on the premise that (1) severity of clinical disease increases with age and (2) that after one infection, the probability that subsequent infections result in disease is reduced [25].

We noted that higher prevalence of *T. bicornis* (odds ratio, 2.502) being detected in white rhinoceros than in black species (Table 1) indicating a species effect. However, we did not find significant effect associating species with prevalence, suggesting that white rhinoceros, even though more susceptible, is not a risk factor to black rhinoceros prevalence. Our result show that the Kenyan white rhino has higher prevalence of *T. bicornis* (66%) compared to 32.1% - 46.6% in the South African populations [13,26]. The high prevalence of *T. bicornis* in white rhinoceros suggests they are important hosts in the epidemiology of this piroplasm. On the contrary, according to a theory postulated by Schmidt & Ostfeld, [27] we suggest that white rhinoceros could benefit black rhinoceros by acting as ‘sinks’ for rhinocerotid piroplasms.

Further, our results show that prevalence among the age-groups of rhinoceros did not differ significantly (Table 1) contrary to the infection pattern in white rhinoceros population in South Africa in which female sub-adults had significantly higher prevalence [13]. Nevertheless, the age-associated inclination in our result is comparable with that of Govender *et al.*, [13] in that peak infections were observed among sub-adults (Table 1). It is postulated that sub-adult rhinoceros of both sexes are subject to numerous stress-related changes such as, reproductive maturity, courtship, mating and territorial fights [13,28] that may suppress immunity and enhance susceptibility [29,30].

Sex-biased prevalence is observed in many parasitic infections with males having higher prevalence and intensity of infections than their conspecific females [31]. In our results, there was no significant sex-biased difference in prevalence (Table 1) though females (56%) seemed to have higher prevalence than males (45%) an observation that concurs with that of Govender *et al.,* [13]. Factors predisposing females to piroplasm are likely to be comparable with those affecting sub-adult rhinoceros.

**Figure 2:**
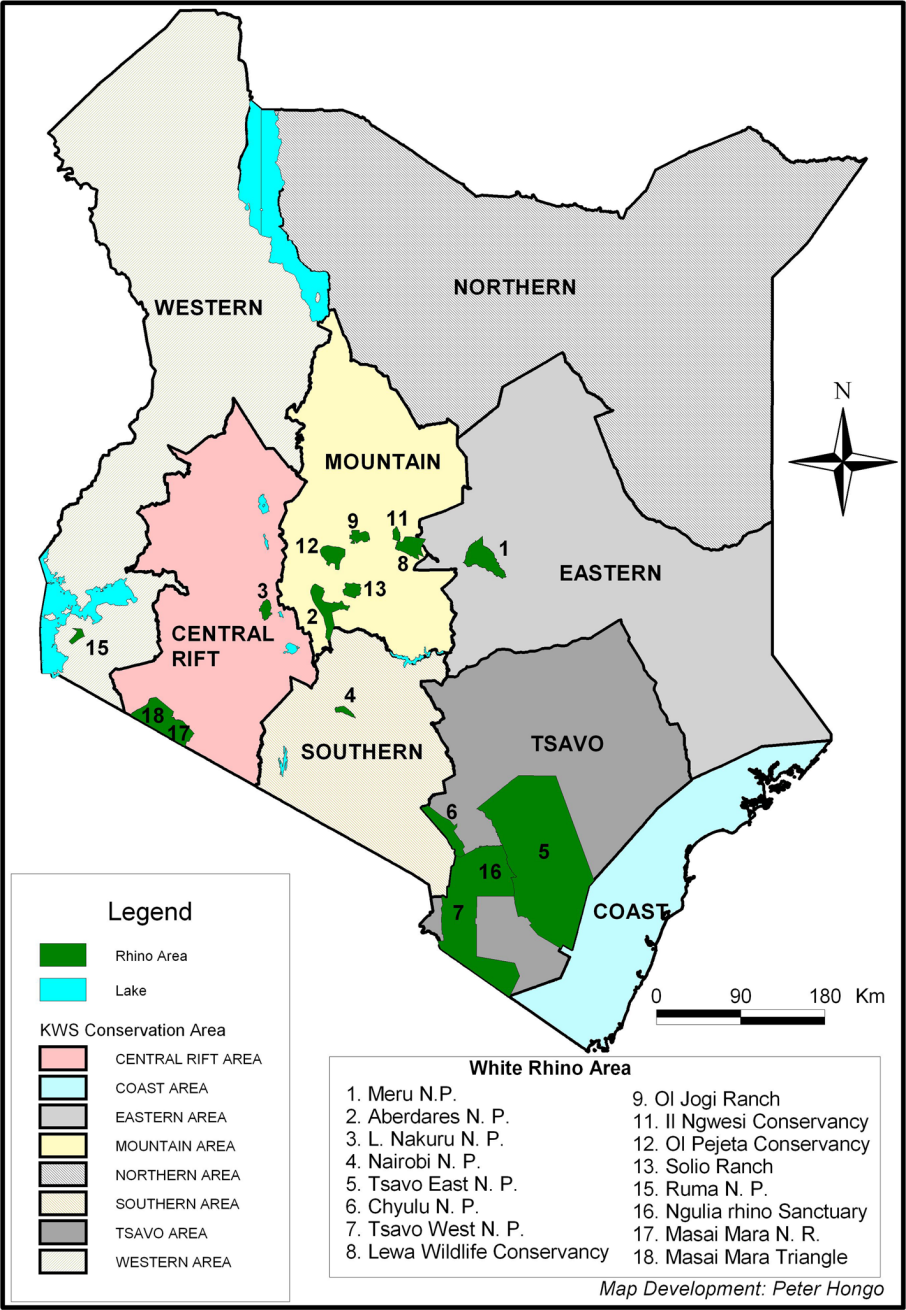
**Locations of black rhinoceros conservation areas in Kenya.**

The occurrence of *T. bicornis* in all the sampled rhinoceros sub-populations could have been facilitated by the regular translocations of individuals. Translocation assists spread of both tick vector and haemoparasites among habitat patches/populations. We observed apparent variations in prevalence among the sub-populations (Figure 2) but there was no significant difference (Table 1). This means that local factors in these habitats, such as ecological and weather differences, mammalian diversity, sanctuary size, were not sufficient to cause significant disparity in prevalence. According to Lopez et al., [32], frequent introduction of parasites in to a patch/habitat via host migration contributes to local patch prevalence. This implies that rhino sanctuaries’ that frequently receive new individuals are likely to harbor higher parasite prevalence. Okita-Ouma et al., [33] points out that LNNP is more of a source population that has supported 41 outward translocations and received one inward translocation, whereas Ngulia RS is a recipient population having received 16 inward translocations and only one outward translocation. However, lack of significant association between location and prevalence (Table 1) does not concur with the postulation of Lopez et al., [32].

## Conclusion

In the analyzed samples we did not detect the presence of *Babesia bicornis*, while *Theileria bicornis* was found to have 49.12% prevalence with white rhinoceros showing a higher prevalence than black rhinoceros. Other factors such as age, sex, location, and population mix were not found to play a significant role.
